# Outbound medical tourism from Mongolia: a qualitative examination of proposed domestic health system and policy responses to this trend

**DOI:** 10.1186/s12913-015-0849-5

**Published:** 2015-05-03

**Authors:** Jeremy Snyder, Tsogtbaatar Byambaa, Rory Johnston, Valorie A Crooks, Craig Janes, Melanie Ewan

**Affiliations:** Simon Fraser University, Blusson Hall 10516, 8888 University Drive, Burnaby, BC V5A 1S6 Canada

**Keywords:** Medical Tourism, Mongolia, Health planning

## Abstract

**Background:**

Medical tourism is the practice of traveling across international boundaries in order to access medical care. Residents of low-to-middle income countries with strained or inadequate health systems have long traveled to other countries in order to access procedures not available in their home countries and to take advantage of higher quality care elsewhere. In Mongolia, for example, residents are traveling to China, Japan, Thailand, South Korea, and other countries for care. As a result of this practice, there are concerns that travel abroad from Mongolia and other countries risks impoverishing patients and their families.

**Methods:**

In this paper, we present findings from 15 interviews with Mongolian medical tourism stakeholders about the impacts of, causes of, and responses to outbound medical tourism. These findings were developed using a case study methodology that also relied on tours of health care facilities and informal discussions with citizens and other stakeholders during April, 2012.

**Results:**

Based on these findings, health policy changes are needed to address the outflow of Mongolian medical tourists. Key areas for reform include increasing funding for the Mongolian health system and enhancing the efficient use of these funds, improving training opportunities and incentives for health workers, altering the local culture of care to be more supportive of patients, and addressing concerns of corruption and favouritism in the health system.

**Conclusions:**

While these findings are specific to the Mongolian health system, other low-to-middle income countries experiencing outbound medical tourism will benefit from consideration of how these findings apply to their own contexts. As medical tourism is increasing in visibility globally, continued research on its impacts and context-specific policy responses are needed.

## Introduction

Medical tourism is an international medical practice where patients travel across national borders with the intention of receiving private medical care. As opposed to state sponsored cross border care arrangements, medical tourists initiate their travel abroad and typically pay for it out of pocket. This practice is gaining attention by policy-makers and health system administrators in high income countries as their citizens are often perceived to be increasingly traveling to low-to-middle income countries for more affordable and accessible care to avoid the high cost of care at home and/or long wait lists for procedures [1]. Medical tourism is not a new practice, however, citizens of low-to-middle income countries have long traveled abroad both regionally and globally to access care not available in their home countries [2].

Outbound medical tourism offers patients the opportunity to access procedures not available or affordable in their home countries, thereby potentially relieving patients of suffering and saving lives [3,4]. Moreover, it is thought that the demand for treatment abroad has the potential to create pressure for improved domestic health systems as patients often return from abroad having received and become accustomed to high quality care [5,6]. However, increased access to medical services abroad through improved travel networks and expansion of the medical tourism sector in many countries has the potential for significant negative economic impacts on patients and their friends and families at home. As lifesaving procedures become within reach through the practice of medical tourism, these patients and their loved ones may lose their savings and incur substantial debt in the pursuit of care that may fail, require expensive follow-up care, or achieve limited success [7,8]. While it has been speculated that the outflow of private patients from these low-to-middle income countries may have ripple effects for their wider economies as well, slowing economic growth and delaying the development of expanded care locally, such implications have yet to be fully examined through on-the-ground empirical research. In this article we address this knowledge gap through considering the ways in which health systems, health policy makers, and clinicians are responding to the practice of outbound medical tourism from Mongolia based on a thematic analysis of 15 face-to-face interviews conducted with stakeholders.

Like many other low-to-middle income countries, Mongolia is seeing a significant outflow of patients to countries such as China, Japan, South Korea, and Thailand for medical care. The reported motivations for Mongolians seeking care abroad include a lack of faith in the domestic health system combined with the hope for treatment afforded by seemingly limitless options abroad [9]. This is consistent with research examining the outflows of patients from other countries, including Yemen and Canada [7,10]. These perceptions are reinforced by the lack of high-technology interventions and reliable diagnostic services of all types in Mongolian health centres [11]. Mongolian patients and their families regularly deplete their savings and impoverish themselves through the purchase of relatively expensive or lengthy treatments abroad, driven by the cultural premium placed on attempting to overcome poor health despite unfavourable odds [9]. As a result, medical tourism from Mongolia has a considerable impact on the Mongolian economy and health system, creating a need to better understand the scope of this practice and practical steps for addressing demand for it. Further to this, there is concern in Mongolia that this practice is having negative economic impacts on the domestic economy and individual finances [9,12]. Expanding on these worries, here we examine the concerns and proposed responses that are developing due to observations or growing awareness of the outflow of patients from Mongolia and their consequent economic effects. While these proposed responses focus on the Mongolian context, they also provide warnings and lessons for other low-to-middle income countries experiencing similar outflows of patients.

## Background

Mongolia is a central Asian country with a population of 2.7 million people and a rapidly growing economy based on resource extraction [13]. Classified as a lower middle-income country, Mongolia’s GNI increased to $3,160 in 2012, up from $1,630 in 2010 [14,15]. Health care is provided through a mix of public and private providers and user fees have been introduced in order to address public funding shortages [16]. In 2002, 25% of total health expenditures case from health insurance and 14% from household budgets in the form of direct payments to providers [16]. Although health care is available by right to all Mongolian citizens, there are serious concerns with the quality of services provided. This is especially true for laboratory and diagnostic services, which is one of the drivers of medical tourism from Mongolia [11,17]. Although both rural and urban areas have experienced shortages in health care personnel, with on average 2.8 physicians per 1000 persons, rural areas are disproportionately affected [18]. This, along with limited access to drugs, diagnostic services, and supplies poses a challenge to rural patients and physicians in particular. Those most affected by these challenges are the vulnerable poor, especially those in urban squatter (‘*ger’*) settlements who face both financial and institutional barriers when accessing health care in Mongolia [19]. Doctors within the public system are allowed to work outside of public facilities and charge fees during off hours and, whereas additional hospital beds have been slow to materialize in the public sector, they are being added much more quickly in the private sector [17]. Low salaries for doctors and nurses have led to the need to work overtime hours and a high level of job dissatisfaction and burnout among these groups [20]. The distribution of health human resources is uneven, with much higher concentrations of workers in the capital Ulan Bataar when compared to rural areas, and rural populations generally have inequitable access to care [17].

Recent economic growth in Mongolia has not translated into better population health. The current main causes of death for adults are noncommunicable diseases such as cerebrovascular disease (14.0%), liver cancer (8.6%) and ischaemic heart disease (6.4%), while injuries and poisoning contribute to youth morbidity [21]. According to the 2009 Mongolian Steps Survey, 62.7% of the population has high blood pressure, less than 7% consume adequate amounts of fruits and vegetables, and just over half of the population is considered overweight or obese [22]. To complicate matters, the country has a large nomadic population distributed across a vast landscale, which impedes efforts to enhance public housing and health care [21]. The large role of extractive industries in Mongolia’s economy has also had an effect on the health of its inhabitants. Mining projects are likely to generate increased in-migration of miners, entrepreneurs, job-seekers, and their families, to areas where employment can be found, placing strain on the existing health system and contributing to the spread of communicable disease [23,24]. The World Bank [25] found that increased mining activity in Mongolia has led to deteriorated water and air quality, as well as mercury and cyanide pollution in the soil and water in particular regions.

The collapse of the Soviet Union in the 1990s significantly affected Mongolia’s economy and health care sector as the country transitioned from a socialist to a capitalist market economy. Prior to this, Mongolia had made large strides in improving the country’s health system, life expectancy had risen substantially, infant and maternal mortality had decreased from improved services, medical and public health infrastructure had been improved, and access to health care was universal [26]. The transition, however, led to a combination of increased privatization, unemployment, and inflation, along with decreased wages and social services [27]. As a part of this economic reform, health care spending declined, resulting in the degradation of existing health care infrastructure [19]. Though compulsory health insurance was introduced in 1994, with government paid premiums for vulnerable populations, there remain populations in need that are excluded and health economists have deemed the national health insurance program unsustainable [28].

Mongolia is an actively sought after source of patients by various medical tourism destinations. International Medical Services Israel (Israel) and Bumrungrad Hospital (Thailand) have recently established recruitment offices in Ulaanbaatar, with Bumrungrad identifying Mongolia as one of its top export markets [9,29]. Mongolia has also been identified as one of seven key markets for ophthalmologic surgery in India [30] and the governments of Korea and Mongolia have established a memorandum of understanding aimed at facilitating the development of the medical tourism industry between the two nations including the transfer of children with cardiac ailments to a Korean hospital [31]. These developments lend credence to reports that medical tourism is rapidly expanding in Mongolia. Estimates of annual patient outflows run as high as 40,000, representing an annual loss to the Mongolian health system of as much as USD$14 million, or 7% of the annual state budget for health care [9,12]. It is known that the Mongolian Minister of Health received 140 formal travel visa requests in 2010 specifically for citizens seeking to travel abroad for medical care, but it is certain that the number traveling informally is much higher [9].

## Methods

In this article we report on the findings of an exploratory study guided by the case study methodology. Case studies require a focus on context in order to understand a phenomenon as it truly occurs, and typically draw on multiple methods [32]. Our methods included face-to-face interviews with health system and policy stakeholders in Mongolia. The analysis presented here draws on the interview data from semi-structured interviews that took place in April, 2012.

### Participant recruitment

Prior to recruitment, ethics approval for the study was obtained from the research ethics board at the investigators’ institution. Local ethics approval was not sought as the researchers were not partnered with a local university or conducting health research under the auspices of the Mongolian Ministry of Health. Following ethics approval, interview participants were purposively sought based on their professional positions and their ability to speak to the issue of medical tourism, excepting the 5 Mongolian medical tourists who were identified through the investigators’ personal networks. E-mails were sent directly to potential participants informing them about the study and requesting their participation in an interview. Interviews were then scheduled with those who were interested in participating. This initial correspondence was conducted in the Mongolian language by TB (a native Mongolian). Everyone who was invited to participate in an interview agreed, resulting in 15 interviews in total. Table 1 summarizes the health system/policy stakeholder roles represented by the participants.

**Table 1:** **Health system/policy stakeholder roles represented by the participants**

**Role**	**Number of Interviewees**
Public Sector Physicians	2
Private Sector Physicians	2
Government Health Administrators	3
Non-Governmental Health Organization Workers	3
Former Medical Tourists	5

### Data collection

Participant consent was sought prior to the interview using a consent form translated into both English and Mongolian. The form presented a review of the study’s goals, the rights of the participant, and potential risks of participation between the researcher and the participant. All interviews were co-conducted using a semi-structured interview guide by JS and TB. One group interview was conducted for which VAC and RJ also attended as observers. This interview followed the same guide as the individual interviews and the interviewer solicited participation from each of the three participants. The interview guide sought to explore participants’ awareness of medical tourism from Mongolia, the push and pull factors that informed Mongolians’ decisions to seek care abroad, and the impacts of medical tourism on the Mongolian health system. The interviews ranged between 30 and 60 minutes. The majority (n = 11) of interviews were conducted in Mongolian with TB working as an interpreter who immediately re-stated responses in English, but a small number of interviews (n = 4) were conducted in English. All interviews were digitally recorded and later transcribed in English by a professional transcriptionist.

### Data analysis

Following transcription, the investigators were each assigned four transcripts to read, two of which were read by all. During independent transcript review, the investigators noted emerging themes and potential codes with which to organize the interview data. The investigators then met to discuss emerging themes from the dataset and resolve any divergent perspectives through seeking consensus on interpretation. The theme of ‘domestic responses to outbound medical tourism’ emerged consistently throughout the transcripts even through questions asked of the participants focused mainly on impacts. In other words, participants were keen to identify the ways in which they were responding to the domestic impacts of outbound medical tourism from Mongolia. Consensus was reached on the interpretation of this thematic finding, after which a coding scheme was developed. The scheme was created using an iterative process that involved amalgamating, discarding, and generating new coding categories that most efficiently and accurately captured the issues present across the transcripts central to the identified theme.

## Results

Thematic analysis of the interviews identified four groups who have roles to play in reducing outbound medical tourism from Mongolia and its impacts through actively responding to this trend through domestic health system and policy reform. These groups serve both as targets for reform and reformers themselves. These groups include Mongolian politicians and government officials (e.g., directors within the Ministry of Health), health workers, health system administrators (e.g., senior officials and policy makers within the Ministry of Health, district health department officials), and patients. In this section we examine these groups separately, while in the discussion section that follows we identify some of the similarities and differences in the policy changes that they propose be undertaken in response to outbound medical tourism from Mongolia.

### Politicians and government officials

The stakeholders we spoke to emphasized the need for politicians and government officials within Mongolia to take steps to make the local health system function more efficiently and fairly as a way to respond to the trend of patients choosing to go abroad for care. In other words, participants indicated that politicians and government officials need to undertake measures that will retain patients domestically. A key concern centered on the need to reduce perceived cronyism and corruption within the system, particularly in the form of favouritism for politically and socially connected patients. One stakeholder said: “*if you don’t know a guy in a hospital, if you don’t know doctors then they will put you through… an inconvenient process and that wait list is so frustrating*.” This need to have personal connections was echoed by another stakeholder, for whom her quality of care was: “*good because my [family member] works at the Ministry. So whenever there is a need we just make personal calls. Otherwise if we just followed the procedures it will take days to get to the proper person*.” For one stakeholder, this cronyism raises issues of “*fairness and [the] issue of having equal access*.” In some cases, this corruption took the form of requiring bribery in order to receive quality service: “*it’s not a legitimate thing, but it’s kind of unwritten requirement that they must reward or bribe everyone including from doctors, nurses and technicians and pretty much everybody who was part of this surgical operation*.” The problem of bribery was not seen as being universal, as *“it really depends on which institution you go [to]*.” Collectively, these issues were thought to push patients abroad and so taking measures to lessen cronyism was thought to be a necessary response to addressing the outflows of Mongolian patients.

Politicians and government officials were accused of not properly managing health resources to limit the demand for medical tourism. For example, many stakeholders spoke of a failure of the government to increase health spending which led to system strain that ultimately pushed patients abroad. In one case, this was connected to the collapse of the Soviet Union where “*we’ve been going through this transition period and because of that lack of money and all this kind of thing …causes weak investment into health sector*.” Increased diagnostic capacity was repeatedly cited as a high priority need for investment. Stakeholders felt that the perceived failure of the government to develop the health sector in Mongolia is a key factor in driving demand for medical tourism and thus a pressing area for reform. For example, one stakeholder spoke of the government’s inability to locate land for a new maternal hospital: “*the political will is not there, so I don’t think Mongolian government is really worried about this medical tourism*.” Another hoped that increased access to private hospitals might reduce usage of medical tourism: “*these new local hospitals, high standard hospitals, are being built and once they complete their project things will get much better. I don’t think an airplane full of patients will go to Bumrungrad [a medical tourism hospital in Thailand]*.” One stakeholder noted that health and education were now priorities for the government and that spending will increase in these areas in the future.

### Physicians

Physicians’ culture of care was cited as a factor discouraging use of the domestic health system and encouraging patients to seek care abroad. Many stakeholders linked the Mongolian culture of care to the old, hierarchical Soviet style of care: “*the style of service we provide in Mongolia is kind of [a] very old Russian style, just perform that procedure and don’t really think about how to get connected mentally with that patient or how to improve our services*.” Instead, it was stated frequently that Mongolian health workers need “*to employ this client friendly service into our daily practices*” in order to retain patients both as a system improvement and also as a direct response to outbound medical tourism. For one stakeholder, this culture of care resulted in health workers not sharing information with their patients: “*the Mongolian doctors and health professionals don’t really explain why what we’re doing…doctor to patient communication needs to be improved*.” A former medical tourist noted that his Mongolian physicians did not “*bother trying to explain*” his test results while “*the Korean doctors took time to explain*.” Medical tourism was cited as a factor in shaping Mongolians’ attitudes toward the culture of care in their own system: “*they love it [medical tourism] because…it sounded like people staying in 5 star hotel and resort while getting treated*.” If the Mongolian domestic system is going to be competitive with international hospitals in the region, “*we probably need to crack a smile when we greet patients*.”

The workload and incentive structure for Mongolian physicians was reported to be problematic. As one stakeholder explained, “*the remuneration system is unfair because first of all they [Mongolian physicians] have very low salary, secondly they have flat salary which has some variation only in terms of…how many years they worked and also whether they have this PhD degree…otherwise it’s not related to their actual performance*.” This same stakeholder noted that the number of patients seen does not factor into salary, meaning there is no incentive to increase efficiency in the public system in response to the loss of patients to other countries. Another stakeholder stated that the performance of Mongolian health workers would be improved through better job satisfaction, “*but we’ve got no system in place to…[provide] benefits to healthcare professionals*.” As another stakeholder put it, Mongolia needs to enable an environment where health professionals “*really want to serve their people better,*” but right now the “*supportive environment is not good*.” Together, the culture of care and failure to reward physician performance are seen as creating a health care environment that is not welcoming to Mongolian patients.

### Administrators

Administrators in the Mongolian health system were seen as important agents in undertaking system responses to reduce outbound medical tourism. One problem noted was a lack of strategic planning around the allocation of those health expenditures approved by the government. The funding allocated to the health system: “*needs to be made based on a rational decision, rational analysis, that way…we’ll be able to increase the efficiency and effectiveness of [the] current system by simply re-allocating those resources*.” Better allocation of resources could be harnessed to address the issue of overly-long wait times that in some cases push patients abroad. As a result, health system administrators need to establish: “*how we then can serve our people better and [in] a timely manner*.” The stakeholders that we spoke to also indicated that there were problems in terms of the management of system resources, including human health resources. One stakeholder indicated that the health system lacks a clear strategy for positioning health human resources within the country: “*how to position, how to maximize the use of this professional skill and knowledge, that's a key issue and there is not really straight and clear strategy how to position our manpower*.” This same stakeholder indicated that this lack of a clear strategy is problematic as “*there is not really [a] registration system or a working system of who deserves what kind of position, what level of position and … how they recruit people and how they fire people*.” These health human resource inefficiencies were seen as undermining attempts to meet the health needs of the Mongolian population, driving outbound medical tourism.

One key problem in the administration of the Mongolian health system that was repeatedly raised was the failure to prioritize investment in training for Mongolian health workers. One stakeholder criticized current health system spending, which subsidizes some care abroad for Mongolians seeking procedures not available domestically: “*why can’t we spend the budget into…trying to help health professionals, trying to build their capacity in order to be sustainable and…to build [the] Mongolian health system capacity as a whole*?” As another put it, the “*core of the problem is the skills of the doctors, increasing the number of procedures and diagnostic methods, they are not doing that. They are just…building new buildings*.” Specialist training was seen as a particular problem: “*they clearly don’t spend enough time to train those people, they’re spending only six months, a standard requires them to at least train for eighteen months and in other countries people do work in residents’ level two to three years*.” Perversely, the failure to invest in human resource training was said to lead to situations where new, highly advanced equipment was going unused or “*in some private hospitals they’re getting more sophisticated equipment but…they do not know how to read the results*.” Because of the low numbers of highly trained specialists, “*if the person goes to another work…then we will be in big trouble finding next person*.” Better allocation of resources by these administrators was thus perceived as a necessary step in reducing the health system inefficiencies pushing Mongolians abroad.

### Patients

As the stakeholders we spoke with observed, significant reforms are needed to the Mongolian health system to reduce the appeal of seeking medical treatment abroad. Many of these stakeholders also noted that perceptions of the Mongolian health system must be improved so that patients are willing to utilize the domestic system. As one former medical tourist noted, when Mongolian doctors told him to drink less alcohol, he did not listen but, “*for some reason I listen to this Korean doctor better*.” Similarly, a stakeholder told us that, “*I lost my faith in [the] Mongolian health system and Mongolian doctors several years ago, so I never go to Mongolian doctors*.” This poor view of the domestic system is in some cases encouraged by the actions of public officials. One stakeholder mentioned a former Minister of Health who “*went to Korea a few times and that was kind of criticized highly through media and…if he’s not trusting the sector that he leads then who’s going to trust it and what does that mean?*” Transforming citizens’ attitudes about domestic health care was thought to be a critical response to stemming the flow of Mongolian patients to other countries. Importantly, one stakeholder took the view that improving public perception of the Mongolian health system would not be an easy task. While some “*professionals try really hard to bring that reputation back to this acceptable level, it doesn’t seem to be working that well. I guess it’s going to take a lot more years than we anticipated. The reputation and people’s faith in the health sector, in our doctors … still don’t seem to be [at the] proper level*.”

In addition to poor perceptions of the system, better information about the services offered domestically was thought to be a needed response to addressing the issue of the outflow of patients to neighbouring countries for private medical care. One stakeholder said that “*in some cases people don’t know…our capacity, that things [high quality treatments] have been offered in Mongolia*.” Similarly, a solution may be to “*simply let the public know that how good Mongolian health system capacity is*.” Unless the public understanding about the services available in the Mongolian health system and public perception of the quality of these services are improved, stakeholder felt that outbound medical tourism was likely to continue at high rates.

## Discussion

The stakeholders we spoke with identified five key areas for reforming Mongolian health policy and Mongolia’s health system that would collectively respond to concern over the domestic impact of outbound medical tourism. First, overall funding of the health system must be increased through effective policy measures, though some stakeholders indicated that increased funding is currently a government priority and thus action to address this concern may be underway. Second, stakeholders urged that existing and future funding be spent more efficiently, including by reducing political influence on spending allocations and targeting high need areas such as diagnostic services. Third, training opportunities for Mongolian health workers should be increased, also targeting areas such as diagnostics. These enhanced training opportunities can increase the quality of care available for patients while increasing health system efficiency by ensuring utilization of existing equipment and other resources. Fourth, incentives and promotions for health workers should be tied to improving system outcomes and efficiency rather than time served in the system as is current practice. Finally, the treatment of patients by health workers should be improved in order to make the Mongolian health system more welcoming. Better communication with patients may also result in improved understanding by health workers of patients’ concerns and increased uptake by patients of health workers’ recommendations for treatment, all of which may lessen patients’ desires to go abroad for medical care. The recommendations focusing on greater and more efficient spending in specific areas such as diagnostic confirms findings in the published literature on the Mongolian health system while concerns about the culture of care in Mongolia are less visible in this literature [11,17].

Patient confidence in the Mongolian health system will likely be increased by the health policy and system improvements proposed above, but the findings show that perceptions that access to care is distributed inequitably also push patients to access care abroad. For patients without the political and professional connections needed to jump the queue for care, medical tourism offers an attractive alternative. Similarly, perceptions that bribery is necessary to access care undermine patient confidence and perpetuate corruption throughout the health system. Mongolian government officials, health administrators, and health workers all have a role in highlighting these issues and taking steps to respond to both the reality and perception of unfairness and corruption in the health system.

Many of the stakeholders we spoke with expressed admiration for the private health facilities abroad that serve Mongolians, noting that they hoped that Mongolians returning home would pressure the domestic system to improve. If this pressure results in greater health system funding and efficiency, increased fairness for health workers and patients, and an improved culture of care, then this pressure should be welcomed. However, some stakeholders took the view that these facilities abroad should be emulated in Mongolia in the form of expanding the private health system there. While private health care is and will continue to be part of the health system in Mongolia, emulating medical tourism facilities will do little to address the basic health needs of most Mongolians, including those without the resources to travel abroad. As these facilities focus on tertiary and specialist care, primary and preventative care will continue to require government support.

While these stakeholders saw expansion of the domestic private health system as a corrective to outbound medical tourism, this proposed response could come at a cost to the public system, including by drawing off health workers and shifting health system priorities [11,33]. These are widespread global concerns linked to medical tourism, and not solely issues for Mongolians or developing economies. Medical tourism has the potential to spread norms of patient care familiar in private settings (e.g., high health worker to patient ratios), that are unaffordable in public systems in high income countries [2]. It can spread care priorities that detract attention from preventative and primary care and public health measures and shift the distribution of health resources according to ability to pay rather than the most pressing health needs of the public [34,35]. These shifts are not inevitable, but must be addressed in all countries with widespread usage of medical facilities abroad. This study of the Mongolian context thus offers important cautions and lessons for other low-to-middle income countries affected by out-bound medical tourism. Given the variety of potential responses to outbound medical tourism identified by these stakeholders, ongoing discussion is needed to determine which steps best meet the needs of the entire Mongolian population.

Additional research into the causes and impacts of outbound medical tourism in Mongolia and other low-to-middle income countries will help to better inform sustainable and equitable responses to this practice. While there is strong evidence that outbound medical tourism from Mongolia and other low-to-middle income countries is having significant negative economic and health system impacts in those countries, information on the number of medical tourists, procedures, sought, destinations traveled to, and costs to these patients is lacking. Data in these areas will strengthen the case for health system reforms and help to direct these reforms toward addressing the specific factors pushing Mongolians and other patients abroad. As Mongolians are actively traveling abroad for medical care and returning to Mongolia with new expectations about quality and cultures of care, research is needed to track changes in service provision in Mongolia, particularly in the private health sector which is likely to be most responsive to these pressures.

### Limitations

One key limitation of this analysis is the small number of Mongolian medical tourists that we spoke with who, unlike the other stakeholder groups interviewed spoke only to their highly individual experiences and perceptions. This small number likely resulted in a restriction of the scope of issues that would have been raised with a larger number of participants from a wider range of backgrounds and experiences of care abroad. The limited number of interviews conducted may also introduce biases that do not reflect the views of Mongolian medical tourism stakeholders at large. Another limitation was the reliance on translation, and especially instantaneous Mongolian-to-English translation during some interviews. While efforts were made to verify that transcripts adequately reflected what was discussed during the interviews, chiefly by ensuring two investigators (CJ and TB) have deep experience and knowledge of Mongolia and its health system and that one of the authors (TB) is fluent in Mongolian and able to confirm the integrity of the translation to the original recordings, having the analysis rely on translated interview data does impose some limits on its accuracy.

## Conclusions

Medical tourism has been observed to have potential negative consequences for the economies and health systems of countries with significant numbers of outbound medical tourists. In this study, 15 Mongolian medical tourism stakeholders were interviewed regarding their perceptions of the causes and impacts of medical tourism from Mongolia. An analysis of the resulting interview transcripts led us to identify four groups (politicians and government officials, physicians, administrators, and patients) with a role to play in effectively responding to the problematic outflow of patients from Mongolia. Proposed health system and policy responses to combat outbound medical tourism include: 1) increasing health system funding; 2) spending existing health system funds more efficiently; 3) increasing health worker training opportunities; 4) tying health worker advancement to performance; and 5) improving the culture of care in the health system. A lack of confidence by Mongolians in their health system was also identified as a primary driver of outbound medical tourism, which must be addressed throughout these five responses. While these findings are specific to the Mongolian context, other low-to-middle income countries are likely facing similar problems from outbound medical tourism. While medical tourism may beneficially aid in driving health system and policy reforms domestically, these countries should be cautious about simply reforming their own health systems to emulate the experience of medical tourists abroad as doing so is unlikely to serve the health needs of the entire population.
